# Mycobacterial cell wall biosynthesis: a multifaceted antibiotic target

**DOI:** 10.1017/S0031182016002377

**Published:** 2016-12-15

**Authors:** KATHERINE A. ABRAHAMS, GURDYAL S. BESRA

**Affiliations:** Institute of Microbiology and Infection, School of Biosciences, University of Birmingham, Edgbaston, Birmingham B15 2TT, UK

**Keywords:** tuberculosis, cell wall, peptidoglycan, arabinogalactan, mycolic acids, antibiotics

## Abstract

*Mycobacterium tuberculosis* (*Mtb*), the etiological agent of tuberculosis (TB), is recognized as a global health emergency as promoted by the World Health Organization. Over 1 million deaths *per* year, along with the emergence of multi- and extensively-drug resistant strains of *Mtb*, have triggered intensive research into the pathogenicity and biochemistry of this microorganism, guiding the development of anti-TB chemotherapeutic agents. The essential mycobacterial cell wall, sharing some common features with all bacteria, represents an apparent ‘Achilles heel’ that has been targeted by TB chemotherapy since the advent of TB treatment. This complex structure composed of three distinct layers, peptidoglycan, arabinogalactan and mycolic acids, is vital in supporting cell growth, virulence and providing a barrier to antibiotics. The fundamental nature of cell wall synthesis and assembly has rendered the mycobacterial cell wall as the most widely exploited target of anti-TB drugs. This review provides an overview of the biosynthesis of the prominent cell wall components, highlighting the inhibitory mechanisms of existing clinical drugs and illustrating the potential of other unexploited enzymes as future drug targets.

## INTRODUCTION

*Mycobacterium tuberculosis* (*Mtb*), the causative agent of tuberculosis (TB), is regarded as the world's most successful pathogen (Hingley-Wilson *et al.* 2003). Responsible for an estimated 1·4 million deaths and 10·4 million new cases of TB, including 480 000 new cases of multi-drug resistant (MDR)-TB in 2015 (World Health Organization, 2016), *Mtb* remains a global health emergency as declared by the World Health Organization (WHO) (World Health Organization, 2014). New chemotherapeutic agents to complement or replace existing front-line treatment regimens are urgently required to reduce treatment time (currently 6-month course) and to combat the increasing threat by this microorganism.

**Fig. 1.**
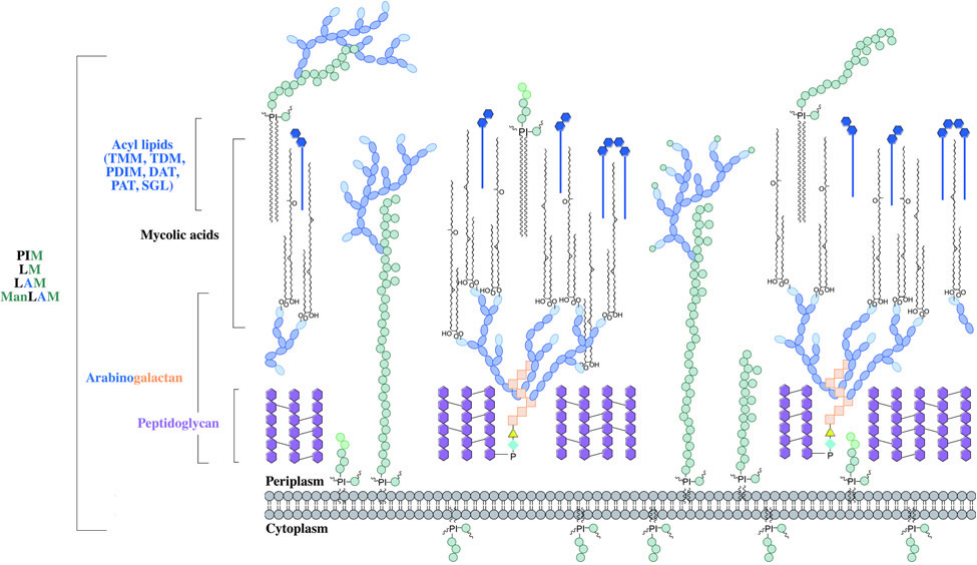
The mycobacterial cell wall. A schematic representation of the mycobacterial cell wall, depicting the prominent features, including the glycolipids (PIMs, phosphatidyl-*myo*-inositol mannosides; LM, lipomannan; LAM, lipoarabinomannan; ManLAM, mannosylated lipoarabinomannan), peptidoglycan, arabinogalactan and mycolic acids. Intercalated into the mycolate layer are the acyl lipids (including TMM, trehalose monomycolate; TDM, trehalose dimycolate; DAT, diacyltrehalose; PAT, polyacyltrehalose; PDIM, phthiocerol dimycocerosate; SGL, sulfoglycolipid). The capsular material is not illustrated.

The distinguishing feature of mycobacteria, the complex cell wall, is a well-recognized drug target. The cell wall is common to all bacteria, both Gram-positive and Gram-negative, but can have vast differences in terms of the biochemical and structural features. Over the past decade, extensive research into cell wall assembly, aided by whole-genome sequencing, has led to an increased understanding of mycobacterial cell wall biosynthesis. This has promoted further exploration into the discovery and development of chemotherapeutic agents (from an enzymatic and phenotypic perspective) directed against the synthesis of this unique macromolecule structure in *Mtb*. The *Mtb* cell envelope is an expansive structure and is summarized in Fig. 1. The inner membrane phospholipid bilayer contains glycolipids that extend into the periplasmic space. The essential core cell wall structure is composed of three main components: a cross-linked polymer of peptidoglycan, a highly branched arabinogalactan polysaccharide, and long-chain mycolic acids. Intercalated into the mycolate layer are solvent-extractable lipids including non-covalently linked glycophospholipids and inert waxes, forming the outer membrane. The capsule forms the outermost layer and is mainly composed of proteins and polysaccharides. The lipid- and carbohydrate-rich layers of the cell wall serve not only as a permeability barrier, providing protection against hydrophilic compounds, but also are critical in pathogenesis and survival. It is these traits that make the biosynthesis and assembly of the cell wall components attractive drug targets. This review focuses on the synthesis of the key cell wall components, highlighting previously validated targets and the ongoing drug discovery efforts to inhibit other essential enzymes in mycobacterial cell wall biosynthesis.

## PEPTIDOGLYCAN

Peptidoglycan is a major component of the cell wall of both Gram-positive and Gram-negative bacteria (Vollmer *et al.* 2008). It is a polymer of alternating *N*-acetylglucosamine and *N*-acetylmuramic acid residues via *β*(1 → 4) linkages with side chains of amino acids cross-linked by transpeptide bridges (Brennan and Nikaido, 1995). Mycobacterial peptidoglycan has a number of unique features that diversifies the cell wall from the typical structure including *N*-glycolyl- and *N*-acetyl-muramic acid residues (Mahapatra *et al.* 2005*a*), amidation of the carboxylic acids in the peptide stems (Mahapatra *et al.* 2005*b*) and additional glycine or serine residues (Vollmer *et al.* 2008). The function of peptidoglycan is not only to provide shape and rigidity, but it is responsible for counteracting turgor pressure and hence it is essential for growth and survival (Vollmer *et al.* 2008). Peptidoglycan is unique to bacterial cells, and it is this property that has led to numerous enzymes involved in its synthesis to be targeted by potent antibiotics, with others representing attractive targets in the development of future antibiotics.

## PEPTIDOGLYCAN BIOSYNTHESIS

**Fig. 2.**
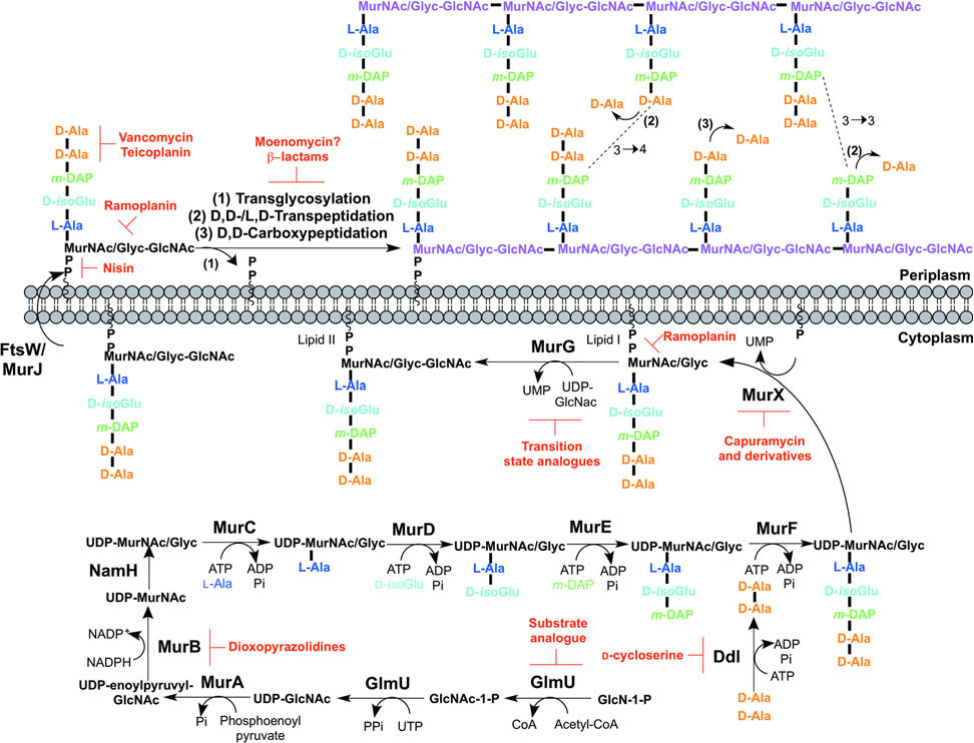
Inhibitors targeting peptidoglycan biosynthesis. The roles of the key enzymes involved in peptidoglycan biosynthesis are illustrated. Reported inhibitors are shown in red.

The biosynthesis of peptidoglycan is summarized in Fig. 2. The first committed step is the generation of uridine diphosphate-N-acetylglucosamine (UDP-GlcNAc). This is catalysed by the acetyltransferase and uridyltransferase activities of GlmU (Zhang *et al.* 2009), where first the acetyl group from acetyl-CoA is transferred to glucosamine-1-phosphate (GlcN-1-P) to produce *N*-acetylglucosamine-1-phosphate (GlcNAc-1-P). Secondly, uridine-5′-monophosphate from UTP is transferred to GlcNAc-1-P to yield UDP-GlcNAc (Zhang *et al.* 2009). The abundance of GlcNAc-1-P in eukaryotes (Mio *et al.* 1998) and the functional similarity of the GlmU uridyltransferase with human enzymes (Peneff *et al.* 2001) makes this domain an unsuitable drug target (Rani and Khan, 2016). However, the absence of GlcN-1-P from humans makes the acetyltransferase domain a potential target (Mio *et al.* 1998). Efforts to identify inhibitors of this domain are underway (Tran *et al.* 2013). A substrate analogue of GlcN-1-P has been designed and exhibits inhibitory effect against GlmU, providing a candidate for further optimization (Li *et al.* 2011).

The next step involves the generation of the UDP-*N*-acetylmuramic acid (UDP-MurNAc)-pentapeptide, which is synthesized in a sequential pathway catalysed by the Mur ligases A–F (Barreteau *et al.* 2008), whereby most of the *Mtb* genes have been found through homology. MurA, a UDP-*N*-acetylglucosamine 1-carboxyvinyltransferase, and MurB, a UDP-*N*-acetylenolpyruvoylglucosamine reductase, are involved in generating UDP-MurNAc from UDP-GlcNAc, by first the addition of the enoylpyruvyl moiety of PEP, followed by reduction to a lactoyl ether moiety via NADPH. At this point, NamH, a UDP-*N*-acetylmuramic acid hydroxylase, hydroxylates UDP-MurNAc to UDP-*N*-glycolylmuramic acid (UDP-MurNGlyc), providing both types of UDP-muramyl substrates; *Mtb* cell walls are dominated by the latter (Mahapatra *et al.* 2005*a*). This structural modification is unique to mycobacteria (and closely related genera) and is considered to increase the intrinsic strength of peptidoglycan, by potentially alleviating susceptibility to lysozyme and providing sites for additional hydrogen bonding (Raymond *et al.* 2005). Inhibitors of *Mtb* MurA and MurB are yet to be discovered. Whilst the natural product, broad spectrum antibiotic, fosfomycin, targets Gram-negative MurA, the critical residue for inhibition is absent in *Mtb*, providing intrinsic resistance against this antibiotic (Kim *et al.* 1996). Consequently, an inhibitor with a new mode of action is required to target *Mtb* MurA. A limited number of inhibitors have been reported against MurB. Molecular dynamics and docking studies of existing MurB inhibitors (3,5-dioxopyrazolidine derivatives) onto the *Mtb* MurB structure reveal the potential potent activity of these compounds, which can be used to guide future structure-based drug design (Kumar *et al.* 2011). Inhibitors of NamH have not been documented; *namH* is not essential in *Mycobacterium smegmatis*, and therefore is not condusive to a characteristic target property. However, gene deletion results in a strain hypersusceptible to *β*-lactam antibiotics and lysozyme and therefore inhibitors of NamH could potentiate the effect of *β*-lactams (Raymond *et al.* 2005).

The pentapeptide chain is incorporated onto the UDP-MurNAc/Glyc substrates by the successive addition of amino acid residues L-alanine, D-isoglutamate, meso-diaminopimelate (*m*-DAP) and D-alanyl-D-alanine [generated by the D-Ala: D-Ala ligase (Ddl)] by the ATP-dependent Mur ligases C-F respectively (Munshi *et al.* 2013). This results in the muramyl-pentapeptide product, UDP-MurNAc/Glyc-L-Ala-D-isoGlu-*m*-DAP-D-Ala-D-Ala, also known as Park's nucleotide (Kurosu *et al.* 2007). Despite the different amino acid specificities, the four ligases share common properties: the reaction mechanism; six invariant ‘Mur’ residues; an ATP-binding consensus; three-dimensional structural domains (Barreteau *et al.* 2008). Due to these similarities, it is plausible that a single inhibitor could target more than one Mur ligase and such inhibitors have been reported in the literature (Tomasic *et al.* 2010). Numerous small molecule inhibitors of the Mur ligases have been discovered and are the subject of an extensive review (Hrast *et al.* 2014). In most cases, the inhibitors were identified from high-throughput screening (HTS) campaigns of compound libraries employing *in vitro* kinetic assays. These types of *in vitro* screening methods are limited in use against *Mtb* Mur ligases given that only MurC and MurE have been biochemically characterized (Mahapatra *et al.* 2000; Li *et al.* 2011). This dictates the next rational step towards the target-based discovery of Mur ligase inhibitors. Ddl is the target of D-cycloserine (Bruning *et al.* 2011), a second-line drug used in the treatment of TB, and is at the cornerstone of treatment for MDR and extensively drug resistant (XDR)-TB. D-cycloserine acts as a structural analogue of D-Ala, inhibiting the binding of either D-Ala to Ddl (Prosser and de Carvalho, 2013*a*, *b*).

The first membrane-anchored peptidoglycan precursor is generated by the translocation of Park's nucleotide to decaprenyl phosphate (C_50_-P), catalysed by MurX (also known as MraY), forming Lipid I (Kurosu *et al.* 2007). There are a number of nucleoside-based complex natural products that inhibit MurX, including muraymycin, liposidomycin, caprazamycin and capuramycin (Dini, 2005). Capuramycin and derivatives exhibit killing *in vitro* and *in vivo* and more significantly, analogues of capuramycin have been shown to kill non-replicating *Mtb*, a feature not common to the majority of cell wall biosynthesis inhibitors (Koga *et al.* 2004; Reddy *et al.* 2008; Nikonenko *et al.* 2009; Siricilla *et al.* 2015). Significantly, the analogue SQ641 is in preclinical development (http://www.newtbdrugs.org).

The final intracellular step of peptidoglycan synthesis is performed by the glycosyltransferase, MurG. A *β*(1 → 4) linkage between GlcNAc (from UDP-GlcNAc) and MurNAc/Glyc of Lipid I is formed, leading to the generation of Lipid II, the monomeric building block of peptidoglycan (Mengin-Lecreulx *et al.* 1991). A library of transition state mimics have been designed for *Escherichia coli* MurG, and tested against *Mtb* MurG with partial success, one being the first inhibitor identified against the *Mtb* enzyme (Trunkfield *et al.* 2010).

The enzyme catalysing the translocation of Lipid II across the plasma membrane has been the subject of much debate. To date, there is evidence for two different enzymes with ‘flippase’ activity: MurJ and FtsW (Ruiz, 2008, 2015; Mohammadi *et al.* 2011, 2014; Sham *et al.* 2014). Further biochemical characterization is required to confirm the identification of the ‘flippase’. Inhibitors against this enzyme would be expected to exhibit broad-spectrum activity, targeting a vital activity in all bacteria.

Following translocation across the plasma membrane, Lipid II is polymerized by the monofunctional and bifunctional Penicillin-binding proteins (PBPs) (Sauvage *et al.* 2008). Bifunctional PBPs (PonA1/PBP1 and PonA2/PBP2) possess transglycosylase and transpeptidase domains. The former domain is responsible for linking the disaccharide building blocks of Lipid II to the pre-existing glycan chains (with the concomitant release of decaprenyl pyrophosphate), whereas the latter domain catalyses the formation of the classical (3 → 4) cross-links, between *m*-DAP and D-Ala of the adjacent pentapeptide chains, with the cleavage of the terminal D-Ala. D,D-transpeptidation and D,D-carboxypeptidation is performed by the monofunctional PBPs, both resulting in the cleavage of the terminal D-Ala of the peptide stem (Goffin and Ghuysen, 2002). Only 20% of the cross-links in *Mtb* peptidoglycan are (3 → 4) (Kumar *et al.* 2012). The majority are (3 → 3) links between two tetrapeptide stems, with the release of the fourth position D-Ala (Lavollay *et al.* 2008). This reaction is catalysed by the L,D-transpeptidases, with D,D-carboxypeptidation as a prerequisite activity. The L,D-transpeptidases are structurally unrelated to PBPs, with different active site residues (cysteine and serine, respectively) (Mainardi *et al.* 2005; Biarrotte-Sorin *et al.* 2006). The *β*-lactam antibiotics have been used in the treatment of bacterial infections for nearly a century, and gave rise to the discovery of their target, the PBPs. The L,D-transpeptidases are resistant to most *β*-lactam antibiotics, except the carbapenems (Dubee *et al.* 2012). Until recently, *β*-lactams were not considered for use in the treatment of TB, due to the expression of a broad-spectrum *β*-lactamase, BlaC. However, it has been shown that BlaC is irreversibly inactivated by clavulanic acid, yet hydrolyses carbapenems at a low rate (Hugonnet *et al.* 2009). Combined treatment of the *β*-lactam with the *β*-lactamase inhibitor has been shown to be bactericidal against both replicating and non-replicating forms of *Mtb*, and combinations are now being explored in clinical trials (Hugonnet *et al.* 2009; Rullas *et al.* 2015). A well-documented inhibitor of the transglycosylase of PBPs, moenomycin (van Heijenoort *et al.* 1987), a natural product glycolipid, is yet to have proven efficacy against *Mtb*.

The inhibitors discussed thus far directly target the enzymes involved in peptidoglycan biosynthesis. There are, however, other antibiotics that act on the peptidoglycan precursors. For example, the glycopeptides, vancomycin and teicoplanin, bind to the D-Ala-D-Ala terminus of the pentapeptide stem, preventing polymerization reactions (Reynolds, 1989). Members of the lantibiotic family of antibiotics, such as nisin, interact with the pyrophosphate moiety of Lipid II, forming a pore in the cytoplasmic membrane, but also inhibiting peptidoglycan biosynthesis (Wiedemann *et al.* 2001). The lipoglycodepsipeptide ramoplanin inhibits the action of MurG by binding to Lipid I. Ramoplanin also binds to Lipid II, preventing its polymerization (Lo *et al.* 2000).

## ARABINOGALACTAN

The major cell wall polysaccharide, arabinogalactan (Fig. 1), as the name suggests, is composed of galactose and arabinose sugar residues, in the furanose (*f*) ring form (Gal*f*) (McNeil *et al.* 1987). Arabinogalactan is attached to peptidoglycan via a single linker unit (McNeil *et al.* 1990). The galactan component is a linear chain of approximately 30 alternating 5- and 6-linked *β*-D-Gal*f* residues (Daffe *et al.* 1990). Three highly branched arabinan chains, consisting of approximately 30 Ara*f* residues, are attached to the galactan chain (Besra *et al.* 1995). The non-reducing termini of the arabinan chains act as an attachment site for mycolic acids, succinyl and galactosamine (D-GalN) moieties (Draper *et al.* 1997; Bhamidi *et al.* 2008).

## ARABINOGALACTAN BIOSYNTHESIS

**Fig. 3.**
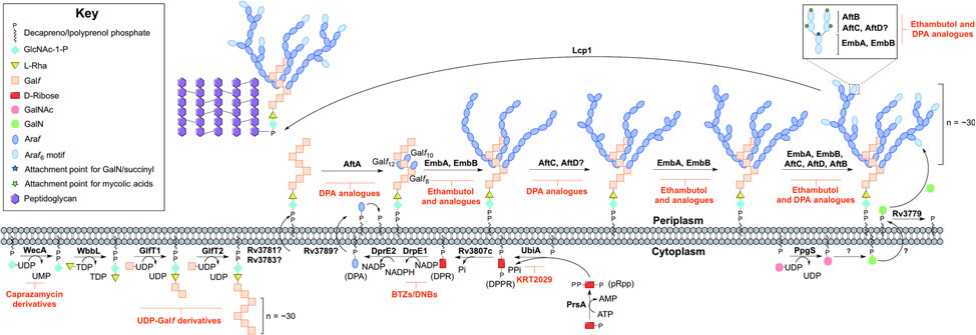
Inhibitors targeting arabinogalactan biosynthesis. The current understanding of the roles of enzymes involved in arabinogalactan biosynthesis. Reported inhibitors are shown in red.

Arabinogalactan biosynthesis is illustrated in Fig. 3. The first committed step begins in the cytoplasm and proceeds by the formation of the linker unit connecting peptidoglycan to arabinogalactan, which is initiated by WecA, a GlcNAc-1-P transferase (Jin *et al.* 2010). This enzyme catalyses the transfer of GlcNAc-1-P to C_50_-P. WbbL, a rhamnosyltransferase catalyses the transfer of L-rhamnose (L-Rha) from dTDP-L-Rha to position 3 of C_50_-P-P-GlcNAc to form C_50_-P-P-GlcNAc-L-Rha, completing the linker unit (McNeil *et al.* 1990; Mills *et al.* 2004). WecA has been identified as the target of caprazamycin derivatives, such as CPZEN-45, with the original nucleoside antibiotic shown to target MraY (Ishizaki *et al.* 2013). Recently, a fluorescence-based assay for WecA activity has been developed and used to screen compound libraries with some success (Mitachi *et al.* 2016). Inhibitors targeting WbbL have yet to be identified. This essential enzyme, present in all mycobacteria, is recognized as a promising target and efforts are underway to characterize the enzyme *via* the establishment of a microtiter plate-based assay for its activity, which could be exploited in inhibitor library screening (Grzegorzewicz *et al.* 2008).

The linker unit provides an attachment point for the polymerization of the galactan chain. This process also occurs in the cytoplasm. The bifunctional galactofuranosyltransferases (GlfT1 and GlfT2) (Alderwick *et al.* 2008) are responsible for the synthesis of the linear galactan chain. Initially, GlfT1 transfers Gal*f* from UDP-Gal*f* to the C-4 position of L-Rha, and then adds a second Gal*f* residue to the C-5 position of the primary Gal*f*, generating C_50_-P-P-GlcNAc-L-Rha-Gal*f*_2_ (Mikusova *et al.* 2006; Alderwick *et al.* 2008; Belanova *et al.* 2008). GlfT2 sequentially transfers Gal*f* residues to the growing galactan chain with alternating *β*(1 → 5) and *β*(1 → 6) glycosidic linkages (Kremer *et al.* 2001*a*; Rose *et al.* 2006). The galactan chains contain ~30 Gal*f* residues *in vivo*, forming C_50_-P-P-GlcNAc-L-Rha-Gal*f*_30_ (Daffe *et al.* 1990), but the chain length determination mechanism is yet to be fully understood. GlfT1 and GlfT2 are suitable targets, as rationalized by an *in silico* target identification program (Raman *et al.* 2008). UDP-Gal*f* derivatives, with modifications to the C-5 and C-6 positions have been investigated as suitable inhibitors of these enzymes, whereby they cause premature galactan chain termination (Peltier *et al.* 2010).

The remainder of arabinogalactan synthesis occurs on the outside of the cell. Although the transport mechanism of this cell wall polysaccharide is not fully understood, Rv3781 and Rv3783, encoding an ABC transporter, are potential ‘flippase’ candidates (Dianiskova *et al.* 2011). Ara*f* residues are transferred directly onto C_50_-P-P-GlcNAc-L-Rha-Gal*f*_30_ from the lipid donor decaprenylphosphoryl-D-arabinose (DPA) (Wolucka *et al.* 1994). DPA is synthesized through a series of cytoplasmic steps, and originates exclusively from phospho-*α*-D-ribosyl-1-pyrophosphate (pRpp), prior to reorientation to the extracellular face of the plasma membrane. The pRpp synthetase, PrsA, catalyses the transfer of pyrophosphate from ATP to C-1 of ribose-5-phosphate, forming pRpp (Alderwick *et al.* 2011*b*). A decaprenyl moiety is added, catalysed by UbiA (decaprenol-1-phosphate 5-phosphoribosyltransferase), forming decaprenol-1-monophosphate 5-phosphoribose (Alderwick *et al.* 2005; Huang *et al.* 2005, 2008). Rv3807c encodes a putative phospholipid phosphatase, which catalyses C-5 dephosphorylation, generating decaprenol-1-phosphoribose (DPR) (Jiang *et al.* 2011). Finally, DPA is generated by an epimerization reaction of the ribose C-2 hydroxyl, catalysed by a two-step oxidation/reduction activity of the decaprenylphosphoribose-2′-epimerase consisting of subunits DprE1 and DprE2 (Mikusova *et al.* 2005).

The DPA synthetic pathway is a validated drug target. The nitro-benzothiazinones (BTZs) and the structurally related dinitrobenzamides target DprE1 and are effective against MDR and XDR strains of *Mtb* with low toxicity (Christophe *et al.* 2009; Batt *et al.* 2012; Makarov *et al.* 2014, 2015). The success of these compounds has led to the study of the other enzymes as potential drug targets. Conditional knockdown mutants of *dprE1, dprE2, ubiA, prsA* and *Rv3807c* have proven the essentiality of all except *Rv3807c*, and a target-based whole-cell screen has been developed using these strains of reduced expression levels to identify enzyme-specific inhibitors. Inhibitors targeting a particular enzyme cause increased sensitivity and this was confirmed with BTZ and KRT2029 targeting DprE1 and UbiA, respectively, and can be the subject of future medicinal chemistry efforts (Kolly *et al.* 2014).

The mechanism of DPA reorientation into the periplasm is unknown. The ‘flippase’ was recently considered to be Rv3789, but there is evidence that this protein plays a different role: to act as an anchor protein to recruit AftA (Kolly *et al.* 2015). AftA is the first arabinofuranosyltransferase (AraT), of a predicted six, to commence the addition of arabinose from DPA onto the galactan chain (Alderwick *et al.* 2006). AftA transfers a single Araf residue onto C-5 of *β*(1 → 6) Gal*f* residues 8, 10 and 12 of C_50_-P-P-GlcNAc-L-Rha-Gal*f*_30_ (Alderwick *et al.* 2005). EmbA and EmbB, so called because their discovery was based on the mode of action elucidation of ethambutol (EMB), catalyse the addition of further *α*(1 → 5) Ara*f* polymerization (Alderwick *et al.* 2005). AftC introduces *α*(1 → 3) branching (Birch *et al.* 2008), with AftD having an equivalent role (Skovierova *et al.* 2009). The structure terminates in a well-defined hexa-arabinofuranosyl (Ara*f*_6_) structural motif: [*β*-D-Ara*f*-(1 → 2)-*α*-D-Ara*f*]_2_-3,5-*α*-D-Ara*f*-(1 → 5)-*α*-D-Ara*f*. This motif is generated by EmbA, EmbB, AftC, AftD and AftB (Escuyer *et al.* 2001; Alderwick *et al.* 2005; Birch *et al.* 2008, 2010; Skovierova *et al.* 2009). AftB catalyses the transfer of the terminal *β*(1 → 2) Ara*f* residues (Seidel *et al.* 2007). C-5 of the terminal *β*-D-Ara*f* and the penultimate 2-*α*-D-Ara*f* of this motif act as anchoring points for mycolic acids (McNeil *et al.* 1991).

The Emb arabinosyltransferases are inhibited by EMB, a well-recognized anti-TB drug, which is employed in the short-course treatment strategy of TB. Efforts are focused on investigating EMB analogues, such as SQ109 (Jia *et al.* 2005*a*, *b*, *c*; Sacksteder *et al.* 2012) and SQ775 (Bogatcheva *et al.* 2006), for future lead drug development. Interestingly, the other AraTs are not inhibited by EMB (Alderwick *et al.* 2006; Seidel *et al.* 2007; Birch *et al.* 2008) and screening for inhibitors against these enzymes is hindered due to the nature of the protein and substrate (membrane bound). However, there have been reports on the development of DPA analogues for the inhibition of arabinogalactan biosynthesis (Pathak *et al.* 2001; Owen *et al.* 2007). A recent study employing a cell free assay approach with membrane preparations has determined that various DPA analogues are able to limit the incorporation of a radiolabelled DP[^14^C]A (Zhang *et al.* 2011).

The primary structure of arabinogalactan is completed by the transfer of succinyl and D-GalN residues to the inner arabinan units. PpgS, polyprenyl-phospho-*N*-acetylgalactosaminyl synthase, catalyses the formation of polyprenol-P-D-GalNAc from polyprenyl-P and UDP-GalNAc, which is then translocated across the membrane (Skovierova *et al.* 2010; Rana *et al.* 2012). The deacylation to polyprenol-P-D-GalN occurs in an undetermined location and by an unknown mechanism. The glycosyltransferase, Rv3779, transfers D-GalN to arabinogalactan at the C-2 position of 3,5-branched Ara*f* residue (Scherman *et al.* 2009; Skovierova *et al.* 2010; Peng *et al.* 2012; Rana *et al.* 2012). Succinylated Ara*f* residues have also been detected at this position of non-mycolated arabinan chains (Bhamidi *et al.* 2008), but the enzyme responsible is currently unknown. A comprehensive mechanistic and functional understanding of these enzymes is required for evaluation as suitable drug targets and to date, there are no identified inhibitors against these processes. The final stage is the attachment of the arabinogalactan macromolecule to peptidoglycan. The enzyme responsible for this essential ligation has recently been elucidated to be Lcp1 (Harrison *et al.* 2016).

## PHOSPHATIDYL-MYO-INOSITOL MANNOSIDES, LIPOMANNAN AND LIPOARABINOMANNAN

The glycolipids, phosphatidyl-*myo*-inositol mannosides (PIMs), and the related lipoglycans, lipomannan (LM) and lipoarabinomannan (LAM), are non-covalently anchored into the inner and outer membranes of the cell wall *via* the phosphatidyl-*myo*-inositol unit (Ortalo-Magne *et al.* 1996) (Fig. 1). The core structure of PIM consists of an acylated *sn*-glycerol-3-phospho-(1-D-*myo*-inositol), the phosphatidyl inositol (PI) unit. Glycosylation with mannopyranose (Man*p*) residues at the O-2 and O-6 positions of *myo*-inositol, results in the mannosyl phosphate inositol (MPI) anchor (Ballou *et al.* 1963; Ballou and Lee, 1964; Nigou *et al.* 2004). The MPI structure is highly diverse, with variations in the type (commonly palmitic and tuberculostearic chains (Pitarque *et al.* 2005)), number and location of acyl chains. The most prevalent forms of PIMs in mycobacteria are tri- and tetra-acylated phospho-*myo*-inositol di/hexamannosides (Ac_1_PIM_2_, Ac_1_PIM_6_, Ac_2_PIM_2_, Ac_2_PIM_6_), where in the hexamannosides, there is one Man*p* unit on the O-2 and five Man*p* units on the O-6 position of *myo*-inositol (Gilleron *et al.* 2001). Extensions of mannan and arabinomannan chains on the MPI anchor form LM and LAM, respectively. In both LM and LAM, the mannan chain consists of approximately 21–34 *α*(1 → 6) linked Man*p* units, decorated with single *α*(1 → 2)-Man*p* residues (Kaur *et al.* 2008). In LAM, the mannan chain is glycosylated through an *α*(1 → 2) linkage with ~50–80 Ara*f* residues (Khoo *et al.* 1996).

In mycobacteria, PI and PIMs contribute up to 56% of all phospholipids in the cell wall and 37% in the cytoplasmic membrane (Goren, 1984). These significant quantities indicate their importance. Not only are they structural components, they also have roles in cell wall integrity, permeability and control of septation and division (Parish *et al.* 1997; Patterson *et al.* 2003; Fukuda *et al.* 2013). LM and LAM are involved in *Mtb* pathogenicity, with evidence to suggest they are modulators of host–pathogen interactions (Schlesinger *et al.* 1994; Nigou *et al.* 2002; Maeda *et al.* 2003). These features of PIMs, LM and LAM make them suitable targets in anti-TB drug discovery.

## BIOSYNTHESIS OF PHOSPHATIDYL-MYO-INOSITOL MANNOSIDES, LIPOMANNAN AND LIPOARABINOMANNAN

**Fig. 4.**
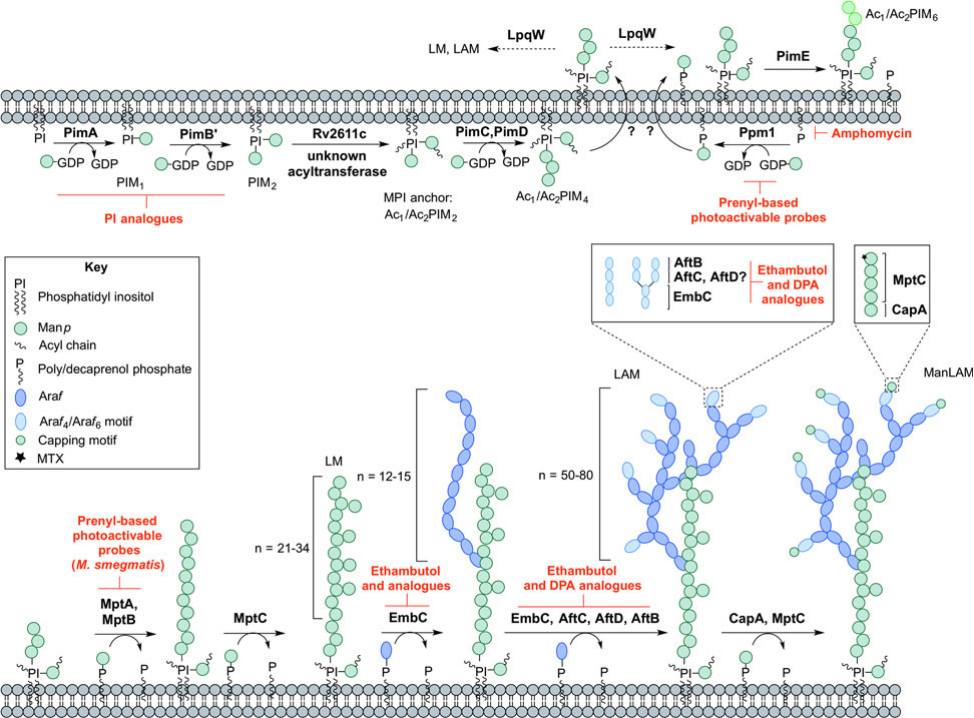
Inhibitors targeting the biosynthesis of phosphatidyl-*myo*-inositol mannosides, lipomannan and lipoarabinomannan. The current understanding of the biosynthesis of PIMs, LM, LAM and ManLAM. Reported inhibitors are shown in red.

PIM biosynthesis begins in the cytoplasm (Fig. 4). The *α*-mannopyranosyl transferase (Man*p*T), PimA, of the GT-A/B superfamily, transfers Man*p* from the donor GDP-Man*p* to position O-2 of the *myo*-inositol ring to form PIM_1_ (Kordulakova *et al.* 2002; Guerin *et al.* 2007). A second Man*p* residue is transferred to position O-6 of the *myo*-inositol ring by PimB’ to form PIM_2_ (Guerin *et al.* 2009). Acylation of the Man*p* residue of PIM_1_ is performed by the acyltransferase Rv2611c before or after the addition of the second Man*p* residue (Kordulakova *et al.* 2003). The acylation of the C-3 position of the *myo*-inositol ring is performed by an unknown acyltransferase. This finishes the synthesis of the MPI anchor. Mannosylation of Ac_1_/Ac_2_PIM_2_ to Ac_1_/Ac_2_PIM_3_ is performed by a Man*p*T, designated PimC, but this enzyme is yet to be confirmed in *Mtb* H37Rv (Kremer *et al.* 2002*b*). It is suspected that the subsequent addition of Man*p* to the non-reducing end of Ac_1_/Ac_2_PIM_3_ is performed by the unidentified PimC or PimD forming Ac_1_/Ac_2_PIM_4_. The Man*p*Ts have been the subject of target-based screening programs. More specifically, *in vitro* PimA activity was screened with approximately 350 compounds. Several hit molecules exhibited significant inhibition, but the compounds did not exhibit *in vivo* activity in *Mtb* (Sipos *et al.* 2015). Substrate analogues of PimA and PimB’, galactose-derived phosphonate analogs of PI, have also been developed, which show enzyme inhibition in a cell-free system (Dinev *et al.* 2007).

The biosynthesis of Ac_1_/Ac_2_PIM_4_ marks the transition towards the synthesis of higher order PIMs, LM and LAM (Fig. 4). It is predicted that the synthesis of Ac_1_/Ac_2_PIM_4_ occurs on the cytoplasmic side of the membrane, and at this point, is flipped across the membrane by an unidentified translocase, with the remainder of the steps thought to occur in the periplasmic space. The integral membrane Man*p*Ts (of the GT-C glycosyltransferase superfamily) are reliant on polyprenyl-phosphate-based mannose donors (PPM) rather than the nucleotide-based sugars (Berg *et al.* 2007). The polyprenol monophosphomannose synthase, Ppm1, catalyses the synthesis of PPM from GDP-Man*p* and polyprenol phosphates (Gurcha *et al.* 2002).

PimE catalyses the transfer of an *α*(1 → 2)-linked Man*p* residue onto Ac_1_/Ac_2_PIM_4_, generating Ac_1_/Ac_2_PIM_5_ (Morita *et al.* 2006). The transfer of the last Man*p* residue is either performed by PimE or by an unidentified GT-C glycosyltransferase forming Ac_1_/Ac_2_PIM_6_ (Morita *et al.* 2006). The distal 2-linked Man*p* residues are not present in the mannan core of LM or LAM; Ac_1_/Ac_2_PIM_4_ is the likely precursor for the extension of the mannan chain. Recent evidence suggests that the putative lipoprotein LpqW channels intermediates such as Ac_1_/Ac_2_PIM_4_ towards either PimE (to form the polar lipids) or to LM and LAM synthesis (Crellin *et al.* 2008). The mannosyltransferases, MptA and MptB (Mishra *et al.* 2007, 2008), are responsible for the *α*(1 → 6)-linked mannan core of LM and LAM. MptC catalyses the transfer of the monomannose side chains via *α*(1 → 2) linkages, forming mature LM (Kaur *et al.* 2008; Mishra *et al.* 2011). Modification of LM leads to LAM. Approximately 50–80 Ara*f* residues are added using DPA as the donor, comparable to that of the arabinogalactan domain. An unidentified Ara*f*T primes the mannan chain, which is further elongated by EmbC, adding 12–16 Ara*f* residues with *α*(1 → 5) linkages (Shi *et al.* 2006; Alderwick *et al.* 2011*a*). AftC, the same enzyme involved in arabinogalactan synthesis, integrates *α*(1 → 3) Ara*f* branches (Birch *et al.* 2008). It has also been speculated that AftD introduces *α*(1 → 3) Ara*f*, but its function is yet to be confirmed (Skovierova *et al.* 2009). The arabinan domain is terminated by *β*(1 → 2) Ara*f* linkages, predicted to be performed by AftB, resulting in branched hexa-arabinoside or linear tetra-arabinoside motifs. Further structural heterogeneity is introduced by capping motifs. These moieties consist of a number of *α*(1 → 2)-linked Man*p* residues, producing mannosylated LAM (ManLAM) (Kaur *et al.* 2008). Using PPM, the *α*(1 → 5) Man*p*T, CapA, attaches the first Man*p* residue (Dinadayala *et al.* 2006). MptC catalyses the addition of subsequent *α*(1 → 2) Man*p* residues (Kaur *et al.* 2008), which can be decorated with an *α*(1 → 4)-linked 5-deoxy-5-methyl-thio-xylofuranose (MTX) residue (Ludwiczak *et al.* 2002; Turnbull *et al.* 2004). The enzymes involved in the addition of MTX and succinyl residues to LAM are still to be determined.

The essentiality of PPM in lipoglycan biosynthesis makes Ppm1 an attractive drug target. Amphomycin, a lipopeptide antibiotic, inhibits the synthesis of PPM by sequestering the polyprenol phosphates, and consequently inhibits the extracellular Man*p*Ts (Banerjee *et al.* 1981; Besra *et al.* 1997). Guy *et al*. (2004) designed a variety of prenyl-based photoactivable probes. Upon photoactivation, a number of the probes exhibited inhibitory activity against *Mtb* Ppm1 and *M*. *smegmatis α*(1 → 6) Man*p*Ts (Guy *et al.* 2004). Substrate analogues of the Man*p*Ts have been designed to investigate enzyme–substrate interactions and mechanisms of action (Brown *et al.* 2001; Tam and Lowary, 2010). These types of studies will provide an invaluable insight into the interactions involved and for the future design of inhibitors.

## MYCOLIC ACIDS

The final distinctive component of the mycobacterial cell wall is the unique fatty acids, termed the mycolic acids (Fig. 1). These unique long chain *α*-alkyl-*β*-hydroxy fatty acids (comprised a meromycolate chain of C_42_–C_62_ and a long saturated *α*-chain C_24_–C_26_) are attached to the arabinogalactan layer, but also make up other outer cell envelope lipids such as trehalose mono/di-mycolates and glucose monomycolate. There are three subclasses of mycolic acids: *α*-mycolates, containing cyclopropane rings in the *cis*-configuration; methoxy-mycolates and keto-mycolates containing methoxy or ketone groups, respectively, and have cyclopropane rings in the *cis*- or *trans*- configuration (Brennan and Nikaido, 1995; Watanabe *et al.* 2001, 2002). Mycolic acids contribute to the permeability of the cell wall, and as such are essential for cell viability, and are also essential in virulence, making the biosynthesis of mycolates suitable drug targets (Liu *et al.* 1996).

## MYCOLIC ACID BIOSYNTHESIS

**Fig. 5.**
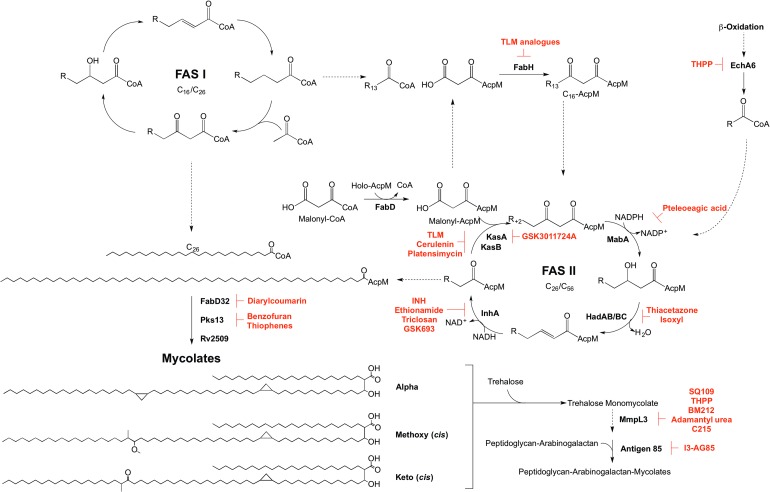
Inhibitors targeting mycolic acid biosynthesis. The enzymes involved in the mycolic acid biosynthetic pathway are presented. Reported inhibitors are shown in red. ‘R’ represents an acyl chain of varying carbon units in length.

Mycolic acid biosynthesis occurs in the cytoplasm, involving two distinct pathways, termed fatty acid synthase types I and II (FAS I and FAS II) (Fig. 5). FAS I (Rv2524c), a multifunctional polypeptide, generates short-chain fatty acyl-CoA esters that can either form the saturated *α*-branch (C_24_), or be extended by FAS II to form the meromycolate chain (Cole *et al.* 1998). Elongation of the fatty acids is dependent on the availability of holo-AcpM, an acyl carrier protein, and malonyl-CoA. FabD, the malonyl:AcpM transacylase generates malonyl-AcpM (Kremer *et al.* 2001*b*). C_14_-CoA primers from FAS I are condensed with malonyl-AcpM, catalysed by FabH (*β*-ketoacyl ACP synthase) (Choi *et al.* 2000), forming a pivotal link between the FAS I and FAS II pathways. The C_16_-AcpM formed is channeled to the FAS II pathway (Bhatt *et al.* 2007), where it undergoes a round of keto-reduction, dehydration and enoyl-reduction, catalysed by: MabA, a *β*-ketoacyl-AcpM reductase (Marrakchi *et al.* 2002); HadAB/BC, a *β*-hydroxyacyl-AcpM hydratase (Sacco *et al.* 2007); InhA, an enoyl-AcpM reductase (Banerjee *et al.* 1994). Successive cycles ensue, whereby the condensation reaction of FabH is replaced by the activities of KasA and KasB, *β*-ketoacyl synthases (Schaeffer *et al.* 2001; Kremer *et al.* 2002*a*). The AcpM-bound acyl chain extends by two carbon units in each cycle, forming a saturated long-chain meromycolate of C_42_–C_62_, which is subject to modifications such as *cis*-/*trans*-cyclopropanation, and the addition of methoxy and keto groups (Dubnau *et al.* 2000; Glickman *et al.* 2000; Glickman, 2003; Barkan *et al.* 2010). FabD32, a fatty acyl-AMP ligase, activates the meromycolate chain (Trivedi *et al.* 2004) and the subsequent meromycolyl-AMP is linked with the *α*-alkyl-CoA ester, catalysed by Pks13, to generate a *α*-alkyl-*β*-keto-mycolic acid (Gande *et al.* 2004; Portevin *et al.* 2004). Finally a reduction step, catalysed by Rv2509, generates a mature mycolate (Bhatt *et al.* 2008). Transport of the mycolates to either the cell envelope or for attachment to arabinogalactan remains to be elucidated. It is considered that the mycolates are transported in the form of trehalose monomycolate (TMM). In the generation of TMM, Takayama *et al*. (2005) propose that a mycolyltransferase transfers the mycolyl group from mycolyl-Pks13 to D-mannopyranosyl-1-phosphoheptaprenol (Besra *et al.* 1994). The mycolyl group of mycolyl-D-mannopyranosyl-1-phosphoheptaprenol is transferred to trehalose-6-phosphate by a second mycolyltransferase, forming TMM-phosphate. The phosphate moiety is removed by a trehalose-6-phosphate phosphatase, and the TMM is immediately translocated outside of the cell using a resistance-nodulation-division (RND) family of efflux pumps, termed mycobacterial membrane proteins large (MmpL), limiting TMM accumulation in the cytoplasm (Takayama *et al.* 2005; Grzegorzewicz *et al.* 2012; Varela *et al.* 2012). Finally, the mycolyltransferase Antigen 85 complex, formed of Ag85A, Ag85B and Ag85C, attaches the mycolic acid moiety from TMM to arabinogalactan (Jackson *et al.* 1999). This complex also catalyses the formation of trehalose dimycolate, TDM, from two TMM molecules with the release of trehalose (Takayama *et al.* 2005). TDM, or ‘cord factor’, is implicated in the pathogenicity of *Mtb*.

The enzymes involved in mycolic acid biosynthesis are the targets of numerous inhibitors. In 1952, shortly after its discovery, isoniazid (INH) was administered as a front-line and essential antibiotic in the treatment of TB (Medical Research Council, 1952) and has only recently had the mode of action elucidated. Initially thought to target KatG due to mutations in the corresponding gene in resistant isolates (Zhang and Young, 1994; Rouse and Morris, 1995), INH was later revealed to be a pro-drug, with the true target being InhA (Banerjee *et al.* 1994; Larsen *et al.* 2002). Ethionamide, a structural analogue of INH, also requires cellular activation via EthA, before targeting InhA (Banerjee *et al.* 1994). Direct inhibitors of InhA that do not require activation are now being searched for (Lu *et al.* 2010; Vilcheze *et al.* 2011; Pan and Tonge, 2012; Encinas *et al.* 2014; Manjunatha *et al.* 2015; Sink *et al.* 2015; Martinez-Hoyos *et al.* 2016). One such molecule is the broad-spectrum antibiotic triclosan, which has not been adopted in TB treatment due to its sub-optimal bioavailability (Wang *et al.* 2004). In the last year, GlaxoSmithKline have published a set of thiadiazole compounds, which directly target InhA, with GSK693 demonstrating *in vivo* efficacy comparable to INH (Martinez-Hoyos *et al.* 2016). Therefore, old drug targets should not be discounted in the search for new anti-tubercular agents.

The *β*-ketoacyl synthases, KasA and KasB, are the targets of the natural products cerulenin (Parrish *et al.* 1999; Schaeffer *et al.* 2001; Kremer *et al.* 2002*a*), platensimycin (Brown *et al.* 2009), and thiolactomycin (TLM) (Kremer *et al.* 2000; Schaeffer *et al.* 2001). There has been significant interest in TLM due to its broad-spectrum activity and numerous analogues have been synthesized to improve on potency and pharmacokinetic properties (Kremer *et al.* 2000; Senior *et al.* 2003, 2004; Kim *et al.* 2006). The biphenyl-based 5-substituents of TLM also exhibit *in vitro* activity against FabH, but with no whole-cell activity (Senior *et al.* 2003, 2004). The 2-tosylnaphthalene-1,4-diol pharmacophore of TLM also has *in vitro* activity against FabH, however, whole-cell data are yet to be published (Alhamadsheh *et al.* 2008). Recently, a new anti-TB compound, an indazole sulfonamide GSK3011724A, was discovered from a phenotypic whole-cell HTS (Abrahams *et al.* 2016). The compound was shown to target KasA specifically, with no discernable target engagement with KasB or FabH, and is currently the focus of medicinal chemistry optimization (Abrahams *et al.* 2016).

Due to the success of InhA as a chemotherapeutic target, there is a mounting interest in the other enzymes involved in mycolic acid biosynthesis from a drug target perspective that could bypass INH resistance in MDR and XDR-TB. Formerly used in the treatment of TB, the thiocarbamide-containing drugs, thiacetazone and isoxyl, were shown to target mycolic acid biosynthesis and the inhibition mechanism has recently been elucidated. Following activation by EthA, both drugs target the HadA subunit of the HadABC dehydratase, forming a covalent interaction with the active site cysteine (Grzegorzewicz *et al.* 2015). It has also been shown that thiacetazone inhibits cyclopropanation of mycolic acids (Alahari *et al.* 2007). MabA has been the subject of a molecular docking study. Comparable with the control inhibitory substrate isonicotinic-acyl-NADH, pteleoellagic acid had a high docking score with *in vivo* activity to be confirmed (Shilpi *et al.* 2015). Through a target-based screening approach linked with whole-genome sequencing of resistant mutants, a benzofuran has been shown to target Pks13 (Ioerger *et al.* 2013). Additionally, Pks13 is the target of thiophene compounds (Wilson *et al.* 2013) including 2-aminothiophenes (Thanna *et al.* 2016). From a GFP reporter-based whole-cell HTS, a diarylcoumarin exhibited potent activity against *Mtb* and this structural class was shown to target FadD32 by inhibiting the acyl–acyl carrier protein synthetase activity (Stanley *et al.* 2013). The homologue of the Rv2509 reductase in *M. smegmatis* is non-essential but loss of function increases susceptibility to lipophilic antibiotics such as rifampicin. Targeting this ‘secondary’ drug target in *Mtb* could increase the susceptibility of the bacilli to antibiotics (Bhatt *et al.* 2008). The Antigen 85 complex has been the focus of a number of inhibitor-based screening studies (Belisle *et al.* 1997; Gobec *et al.* 2004; Sanki *et al.* 2008, 2009; Elamin *et al.* 2009; Barry *et al.* 2011). Recently, an inhibitor from a compound library was shown to bind to Antigen 85C, and derivatives of this compound have been synthesized, with 2-amino-6-propyl-4,5,6,7-tetrahydro-1-benzothiphene-3-carbonitrile (I3-AG85) exhibiting the lowest MIC in *Mtb* and drug-resistant strains (Warrier *et al.* 2012).

In the target identification of new anti-tubercular compounds, some targets can be regarded as promiscuous, inhibited by multiple different chemical scaffolds, exemplified by MmpL3 (Grzegorzewicz *et al.* 2012; La Rosa *et al.* 2012; Stanley *et al.* 2012; Tahlan *et al.* 2012; Lun *et al.* 2013; Remuinan *et al.* 2013), a predicted TMM transporter. Through the generation and sequencing of spontaneous resistant mutants, a number of inhibitors with diverse chemical structures have been shown to target MmpL3 (Grzegorzewicz *et al.* 2012; La Rosa *et al.* 2012; Stanley *et al.* 2012; Tahlan *et al.* 2012; Lun *et al.* 2013; Remuinan *et al.* 2013). However, a recent chemoproteomics approach determined that one of the proposed inhibitor classes of MmpL3, the tetrahydropyrazo[1,5-a]pyrimidine-3-carboxamides (THPPs), has a novel alternative target, EchA6 (Cox *et al.* 2016). Sequence analysis predicted EchA6 to be an enoyl-CoA hydratase, but it lacks the residues required for catalytic activity. Through an extensive biochemical investigation, Cox *et al*. (2016) predicted that EchA6 shuttles fatty acyl-CoA esters from the *β*-oxidation pathway into FAS II, ready for the condensation activities of KasA or KasB with malonyl-AcpM. This research demonstrates that target identification of inhibitory compounds can unveil not only a new biological pathway, but also an untapped area for drug targets.

## DRUG DISCOVERY EFFORTS

The strategies involved in drug discovery are forever evolving. Traditional enzyme screening campaigns and medicinal chemistry focused on ligand-based inhibitor designs (such as substrate or transition state analogues) that once dominated drug discovery are being superseded by phenotypic HTS. The former approach often relies on the X-ray crystal structure of the enzyme or biochemical understanding, and successful inhibitors from these screens are further challenged by target engagement *in vivo.* Over recent years, HTS has become the lead approach in drug discovery. HTS employs extensive compound libraries of diverse chemical structures, and as a consequence, these methods can identify a multitude of inhibitors with novel chemical scaffolds. Phenotypic HTS can reveal anti-TB agents with whole-cell activity and unknown modes of action, having the potential to unveil new biochemical pathways (Abrahams *et al.* 2012, 2016; Gurcha *et al.* 2014; Mugumbate *et al.* 2015). Alternatively, phenotypic HTS can be target-based, focusing on enzymes or pathways such as those involved in cell wall biosynthesis. This can be a very effective way to identify novel anti-TB compounds with known modes of action, but is limited by the specified target (Batt *et al.* 2015; Martinez-Hoyos *et al.* 2016). Target assignment is a fundamental step in in the drug discovery pipeline. Without knowledge of the physiological target, efforts can be wasted on developing compounds against an unsuitable target, such as those homologous in humans. Establishing the mode of action of an inhibitor is a prerequisite for facilitating medicinal chemistry efforts to convert compounds into potential drug candidates.

### Concluding remarks

The essential mycobacterial cell wall, responsible for structural integrity, permeability and pathogenicity, is an attractive drug target, both structurally and biosynthetically. Recent advancements in biochemical and omics-based techniques have led to the discovery and mechanistic understanding of enzymes involved in mycobacterial cell wall synthesis and assembly. Although a number of key enzymes are yet to be established, there are a plethora of suitable targets, exploited not only in current treatment programmes but also for anti-TB drug discovery. In the current TB treatment regimen, two of the front-line drugs, INH and EMB, target mycolic acid and arabinogalactan biosynthesis, respectively, with the second-line drugs such as ethionamide and D-cycloserine also targeting cell wall production. The proven success of these drugs validates the future development of inhibitors targeting the unique mycobacterial cell wall, which remains a source of unexploited clinically relevant drug targets. The continued progression in drug discovery approaches and the optimization of biochemical techniques, will enable the rapid identification of anti-TB agents, many of which are likely to target the biosynthesis of the so-called ‘Achilles heel’ of *Mtb*.
